# 氢键有机框架的制备及其分离分析应用

**DOI:** 10.3724/SP.J.1123.2026.01011

**Published:** 2026-11-08

**Authors:** Shengnan LÜ, Molin LI, Jiahui ZHU, Luliang WANG

**Affiliations:** 鲁东大学食品工程学院，山东 烟台 264025; School of Food Engineering，Ludong University，Yantai 264025，China

**Keywords:** 氢键有机框架, 制备方法, 固定相, 固相萃取, 吸附去除, hydrogen-bonded organic frameworks （HOFs）, synthesis method, stationary phase, solid-phase extraction, adsorption removal

## Abstract

氢键有机框架（hydrogen-bonded organic frameworks，HOFs）是一类由有机构筑单元通过分子间氢键等非共价作用定向组装形成的晶态多孔材料。因其具有结构可调性强、合成条件温和、孔表面易于功能化及骨架可修复等独特优势，在分离分析领域表现出显著潜力。本文总结了HOFs的溶剂挥发法、溶剂热法和自组装制备方法，以及HOFs复合材料的原位生长、化学键合、掺杂共组装和后修饰功能化方法。着重阐述了HOFs在气相色谱和毛细管电色谱固定相，分散固相萃取、固相微萃取和磁固相萃取，以及重金属、放射性核素、有机染料吸附去除方面的应用进展和机理研究。依据上述系统梳理，该综述揭示了HOFs材料的结构与性能关系，同时讨论了HOFs在提升稳定性、调控选择性以及实现规模化制备方面所面临的挑战与应对策略。目的是为HOFs在分离分析领域的深入研究和实际应用提供系统的理论参考与方法学借鉴，从而推动这种新兴材料从基础研究向实际应用跨越。

## Abstract (en)

This review provides a comprehensive and systematic overview of recent advances in the synthesis，functionalization and separation-related applications of hydrogen-bonded organic frameworks （HOFs） and their composite materials. HOFs represent an emerging and distinctive class of crystalline porous materials，constructed from organic building blocks via directional hydrogen bonding and other non-covalent interactions. They exhibit high crystallinity，tunable structures，mild synthesis conditions，ease of regeneration，and solution processability. Owing to these traits，particularly the designable pore environments and modifiable surfaces，HOFs hold great promise for advanced separation tasks that demand precise molecular recognition and efficient mass transfer. The first part of this paper reviews the main design strategies，synthetic methodologies and post-synthetic modification techniques of HOFs. The key preparation methods such as solvent evaporation，solvothermal synthesis and self-assembly，as well as advanced preparation methods such as in-situ growth on substrates，are introduced in detail. The review focuses on the important preparation parameters，underlying separation mechanisms and diverse applications of HOFs. Post-synthesis modification strategies including chemical grafting and functional group conversion have been particularly emphasized. These methods can adjust the properties of HOFs without compromising their structural integrity. This review also explores the mechanisms by which synthesis and improvement methods affect the structural stability，pore properties and functional performance of composite materials. The second part summarizes the various applications of HOF materials in the field of separation science over the past few years. In the application of chromatography solid-phase，the use of HOFs as a new type of stationary phase in gas chromatography and capillary electrochromatography was explored. Their excellent chromatographic separation ability is attributed to their pore structure，uniform pore size，and designable surface functional groups of HOFs. In terms of sample pretreatment，the applications of HOFs and their composites in dispersive solid-phase extraction，solid-phase microextraction and magnetic solid-phase extraction are elaborated. These application methods demonstrate high extraction efficiency，rapid adsorption kinetics and excellent reusability. The application of adsorption removal by HOFs has been comprehensively and deeply summarized. This includes the capture of heavy metal ions，the storage of radioactive nuclides，the recovery of volatile iodine and the removal of organic dyes. These reports indicate that HOFs possess high adsorption capacity，high selectivity and stability under extremely harsh conditions. In the review，it was found that HOFs possess inherent characteristics such as pre-designed framework structure，high specific surface area，modular functions and unique self-repairing properties. These properties determine their separation performance*.* This article clarifies the selectivity，adsorption capacity，kinetic properties and recyclability based on HOFs，highlighting the structure-function relationships. The above systematic analysis provides a methodological reference for designing HOFs to address targeted and challenging separations. In conclusion，because of their designable structures and mild synthesis methods，HOF materials demonstrate great potential for application in separation science. However，challenges such as long-term stability in harsh environments，large-scale production and anti-interference capabilities in real samples still exist. Future research should focus on developing more stable HOFs materials，integrating them into micro-devices and utilizing artificial intelligence-assisted design to accelerate practical applications in environmental protection and resource recovery.

氢键有机框架（hydrogen-bonded organic frameworks，HOFs）是一类新兴的晶态多孔材料，其核心是通过有机构筑单元之间可逆的氢键等非共价相互作用进行定向自组装^［1-7］^。作为多孔晶体骨架材料的重要成员，HOFs与金属有机框架（metal-organic frameworks，MOFs）和共价有机框架（covalent organic frameworks，COFs）共同构成了现代多孔材料研究的前沿^［8-14］^。然而，HOFs凭借其独特的成键本质，展现出迥异于MOFs与COFs的性质，从而在低碳、节能的分离过程及其他诸多功能应用中展现出巨大潜力^［15，16］^。

HOFs、MOFs和COFs的差异源于其构建骨架的核心化学键类型^［17-22］^。MOFs依靠金属离子和有机配体之间较强的配位键连接，COFs则由有机单元通过强共价键连接形成，而HOFs由能量相对较低但高度可逆且具有方向性的氢键驱动组装而成。这种化学键的差异直接决定了三类材料的不同特性。由于氢键具有柔性、可逆性和自愈合特性，HOFs具备一系列MOFs与COFs难以比拟的独特优势：一是合成条件温和，溶液可加工性好，通常在室温或近室温条件下合成，然后通过溶剂挥发、液相扩散等温和方法结晶获得单晶，所得材料能溶于常见有机溶剂，实现溶液状态下的再加工、自修复和再生，为薄膜、涂层及复合膜材料的制备带来很大的便利和灵活性；二是生物相容性好，HOFs多由不含金属中心的纯有机材料构建，部分由金属有机配体构建的材料也选用具有良好生物相容性的金属，避免金属浸出毒性问题，可有效规避MOFs可能存在的风险，使其在生物传感、药物递送和生物医学应用领域具有天然优势；三是理论孔隙率高，以纯有机物为骨架的HOFs去除了重金属，在相同质量下，理论密度更低、潜在孔隙体积更高，有利于提高比表面积；四是结构具有动态响应性，氢键作用力较弱，使得HOFs结构的动态调整更具潜力，其孔道和骨架可对pH、温度、特定气体分子、光等外部刺激产生可逆响应，为开发智能、自适应型分离与传感材料提供了可能^［23-28］^。

HOFs材料的结构稳定性与永久孔隙率的维持，主要依赖于其内部的氢键相互作用。研究发现，如果去除孔道内的溶剂等客体分子，HOFs中氢键作用就会发生重构，进而导致HOFs框架坍塌，孔径和孔隙丧失^［29，30］^。为了解决上述问题，多种协同策略（如引入*π-π*堆积和电荷辅助氢键等次级相互作用、采用具有明确方向性的羧酸二聚体等强氢键基序作为坚固的结构连接点、设计刚性有机构筑单元，限制骨架弛豫并增强抗形变能力）能够有效提高HOFs的稳定性^［1，3］^。利用以上策略，目前已成功制备出热稳定性好、耐沸水且具备良好化学稳定性的多孔HOFs材料^［5，7］^，极大地拓展了HOFs材料的应用领域。在气体分离与储存方面，HOFs具有可定制的微孔环境和表面化学性质，能够通过尺寸筛分和选择性吸附实现气体的高效分离^［31，32］^；在吸附去除中，HOFs材料常用于高效捕获重金属、有机染料等污染物，对环境修复有很大的实用价值^［33］^；在膜分离领域，常用于制备自支撑膜，由于HOFs具有可加工特性，也可以将其作为填料与聚合物基质复合，构建具有高渗透性和选择性的混合基质膜^［34，35］^。由于HOFs具有优异的分离选择性，因此被广泛用作色谱固定相、固相萃取材料等^［36］^。在质子传导、异相催化、化学传感、手性分离以及生物技术与药物递送等其他功能应用领域，针对HOFs的探索也越来越活跃，进一步证明了其作为多功能平台的广泛潜力^［37-39］^。

尽管近年来HOFs在分离分析领域取得了明显进展，但从整体发展水平来看，仍滞后于MOFs和COFs。为了帮助材料化学、分析化学等相关领域的研究者获得更加全面的参考与启发，本文对HOFs的制备方法、功能化策略及其在分离分析中的最新应用进行了较为系统的梳理与总结。

## 1 HOFs的制备策略

HOFs合成的本质是在溶液中利用有机构筑单元自组装形成有序的晶态多孔结构。制备的关键在于控制分子间氢键、*π-π*堆积等弱相互作用的形成与平衡。目前常用的合成手段主要包括溶剂挥发法、溶剂热法和自组装合成法。

### 1.1 溶剂挥发法

溶剂挥发法是最简便温和的合成方法之一。该过程通常将有机配体溶于良溶剂中，在室温或温和加热条件下使溶剂缓慢挥发。随着溶液过饱和度的逐渐提高，有机配体分子通过氢键等非共价相互作用逐步结晶析出。Farajzadeh课题组^［40］^通过溶剂挥发法，利用1，3，5-苯三甲酸（H_3_BTC）成功制备了相关HOFs。该实验将617 mg H_3_BTC加入10 mL甲醇中，超声处理10 min使其完全溶解，随后室温静置24 h，待溶剂自然挥发，得到白色固体产物，研磨后可直接用作分散固相萃取（DSPE）的吸附剂。这一方法通过一步操作便将简单单体转化为具有吸附功能的晶态材料，充分体现了HOFs易于合成的优势。所得材料比表面积仅为2.326 6 m^2^/g，平均孔径15.523 nm，为介孔材料。Luo课题组^［41］^也利用溶剂挥发法合成了手性HOF-2。将手性配体（*R*）-4，4，6，6-四（2，4-二氨基-1，3，5-三嗪-6-基）-2，2-二乙氧基-1，1-联萘溶于2-甲氧基乙醇中，在室温下自然挥发溶剂，直至黄色晶体析出，得到高质量的HOF-2晶体用于气相色谱（GC）固定相。这种方法设备简单，易于操作，适合于热敏性配体，但是结晶时间较长，产物的大小和形状较难精确调控。

### 1.2 溶剂热法

溶剂热法通常是指在密闭反应釜（如聚四氟乙烯内衬的高压釜）中，以水或有机溶剂为介质，在高于溶剂常压沸点的温度下进行反应。高温高压的环境可以提高难溶性配体的溶解度，加速分子运动和重排，有利于形成热力学稳定的晶相，尤其适用于构建刚性较强、需要克服较大能垒的复杂HOFs结构。Jiang课题组^［42］^采用溶剂热法合成了具有八重互锁结构的HOF-BTB。将配体1，3，5-（4-羧基苯基）苯（H_3_BTB）溶于水与乙醇的混合溶剂中，经超声形成均匀的乳白色溶液后，转移至100 ℃的烘箱中反应48 h。冷却后即得到无色粉末状晶体。其比表面积高达1 095 m^2^/g，平均孔径约为1.85 nm。HOF-BTB具有良好的热稳定性（约300 ℃）和化学稳定性。研究证明温和的溶剂热条件为构筑单元提供了足够的能量进行有序自组装，从而形成了结构高度有序且稳定的多孔框架。

### 1.3 自组装合成法

此方法将构筑单元配体的合成与HOFs的结晶过程整合在一个反应体系内连续进行，大大简化了步骤。通过引入催化剂如离子液体可以加速关键成键反应（如亚胺缩合反应），并在温和条件下直接导向大尺寸单晶HOFs的形成，为克级规模合成提供了可能。Wang课题组^［43］^开发了一种在常温常压下克级合成单晶HOFs的策略（见图1）。他们以离子液体［BMIm］［PF_6_］为催化剂，在乙腈溶剂中，使四面体构筑单元四（4-甲醛基苯基）甲烷与对苯二胺或4，4′-二氨基联苯发生亚胺缩合，原位生成四重亚胺连接的五聚体，分别为H1和H2。这些寡聚体随后通过氢键自组装，直接形成大尺寸单晶。该方法将寡聚体合成与HOFs结晶一体化，优化后H1的产率可达83%，并实现了克级规模的制备，突破了单晶HOFs大规模合成的瓶颈。该材料也展现出优异的化学稳定性，能在四氢呋喃、甲苯、甲醇、水、碱性水溶液甚至冰醋酸等多样溶剂中保持晶体结构完整，并且在80 ℃空气中也能保持稳定。

**图1:**
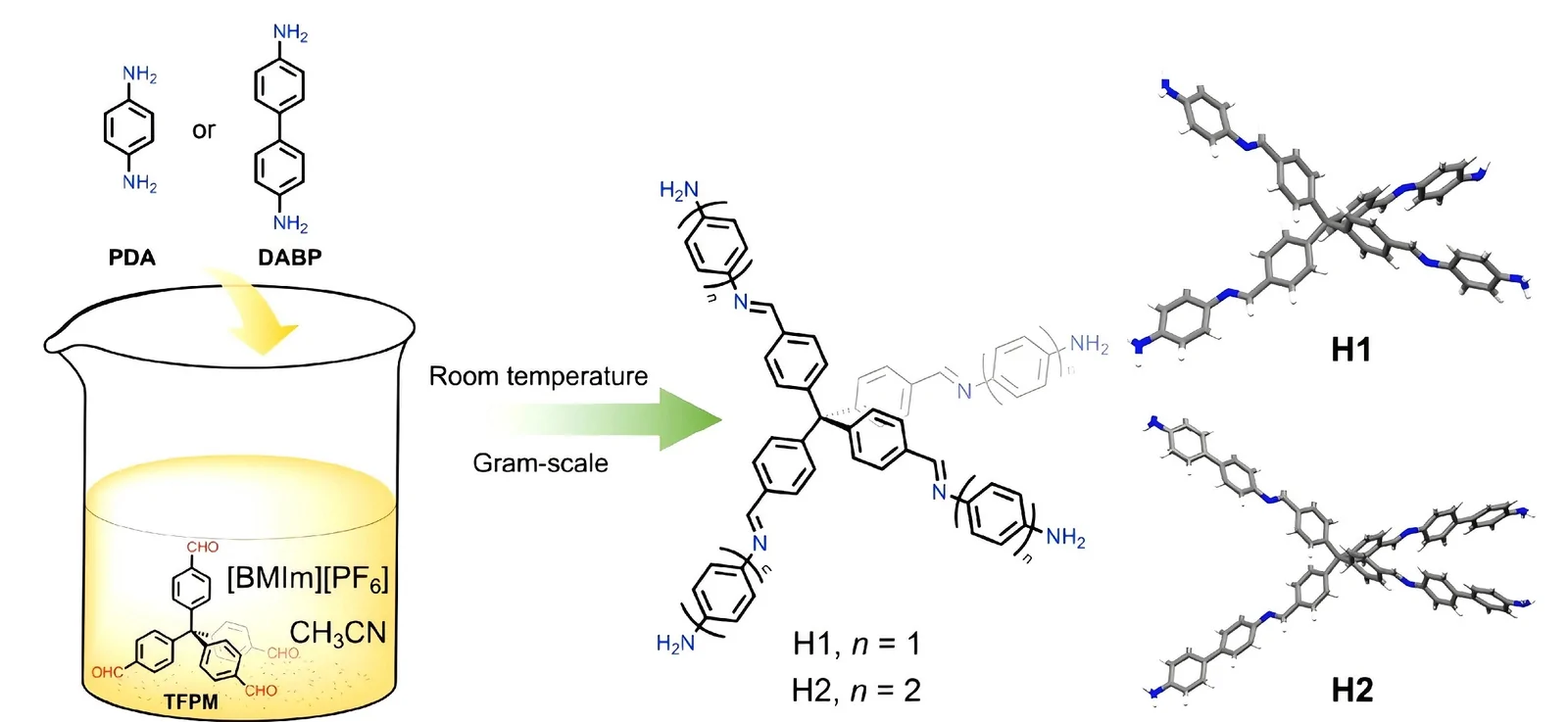
四重亚胺连接的五聚体H1和H2的结构和离子液体催化自组装合成示意图^［43］^ PDA： *p*-phenylenediamine； DABP： 4，4′-diaminobiphenyl； ［BMIm］［PF_6_］： 1-butyl-3-methylimidazolium hexafluorophosphate； TFPM： tetrakis（4-formylphenyl）methane.

为了得到结晶度高、孔隙率适宜、稳定性好并且具有良好形貌的HOFs材料，需重视材料的理性设计和协同作用，在刚性骨架单元选择（例如联苯、卟啉等）与方向性强的氢键配体（如羧酸二聚体）相结合的基础上，还要充分利用*π-π*堆积、电荷辅助氢键及框架互穿等策略，来提升结构的机械强度和化学稳定性。在此基础上还要对结晶过程进行精细动力学调控，在溶剂挥发法制备中，通过调节良/不良溶剂比例、浓度、挥发速度和温度等来控制晶体成核与生长；在溶剂热合成过程中，可以调节反应温度、时间和溶剂种类等参数以获得热力学更稳定、结晶度更高的晶相。另外还需要继续探索创新与规模化合成路径，如一锅法自组装、机械化学及微波辅助合成等手段，从而简化步骤、节省时间并提高产物收率，有利于实现材料的可控合成，为进一步应用于实际生产提供条件。

## 2 HOFs复合材料的制备方法

为拓展HOFs的应用性能（如赋予磁性便于分离、增强导电性等），常将其与其他功能材料复合。制备的关键是如何实现HOFs与基质材料间的稳固结合。

### 2.1 原位生长法

原位生长法是制备核壳结构复合材料最常用的方法之一。在HOFs的形成条件下，使其在核心基质材料表面固定并外延生长，最终形成以基质材料为核、HOFs为壳的复合材料。该方法能实现HOFs壳层的均匀包覆和牢固结合。我们课题组^［44］^通过原位生长法制备了核壳型磁性复合材料Fe_3_O_4_@HOFs。首先合成羧基功能化的Fe_3_O_4_纳米粒子（Fe_3_O_4_-COOH），然后将其分散于含有配体1，3，6，8-四（4-羧基苯）芘的混合溶剂中。在温和的室温条件下，配体可在Fe_3_O_4_球表面自组装生长，形成HOFs壳层。通过优化配体投料量，制备了对四环素具有最大吸附容量的复合材料。该材料兼具HOFs的丰富吸附位点和Fe_3_O_4_的磁响应性，便于磁分离回收。

### 2.2 化学键合法

为了实现更稳定、更均匀的固定，特别是用于开管毛细管电色谱等需要承受液体冲刷的场景，常采用化学键合法将HOFs固定到载体（如毛细管内壁）上。通常先对载体表面进行硅烷化等活化处理，引入氨基等活性基团，再通过共价键或强相互作用将HOFs或经修饰的HOFs键合到表面。Ye课题组^［45，46］^采用了化学键合策略，首先合成Eu-MOF/HOF复合材料，然后用（3-氨丙基）三乙氧基硅烷（APTES）对其进行表面修饰，引入可水解的乙氧基，得到Eu-MOF/HOF-APTES。随后，将经NaOH和HCl活化的毛细管内壁浸入该分散液中，乙氧基水解生成的硅烷醇与毛细管硅羟基发生缩合反应，从而将复合材料以硅氧烷键形式牢固地涂覆在毛细管内壁，制备出稳定性优异的开管毛细管电色谱柱。

### 2.3 掺杂共组装法

在HOFs的合成过程中，直接引入第二种功能分子或构筑单元，使其共同参与自组装过程，形成杂化的复合框架。这种方法可以实现分子水平的均匀复合，有效调控框架的极性、孔径和表面化学性质。Chen课题组^［47］^将三聚氰胺（MA）直接掺杂到HOFs配体1，3，6，8-四（4-羧基苯）芘（H_4_TBAPy）的自组装体系中。MA的加入不仅改变了反应溶剂的极性，使生成的HOFs形貌更均一，更重要的是其丰富的氨基与H_4_TBAPy的羧基形成了更强的氢键相互作用，从而构建了具有更丰富微孔和更高比表面积的HOFs复合材料MA/PFC-1（见图2）。这种共组装策略实现了对HOFs材料性能的精准调控。

**图2:**
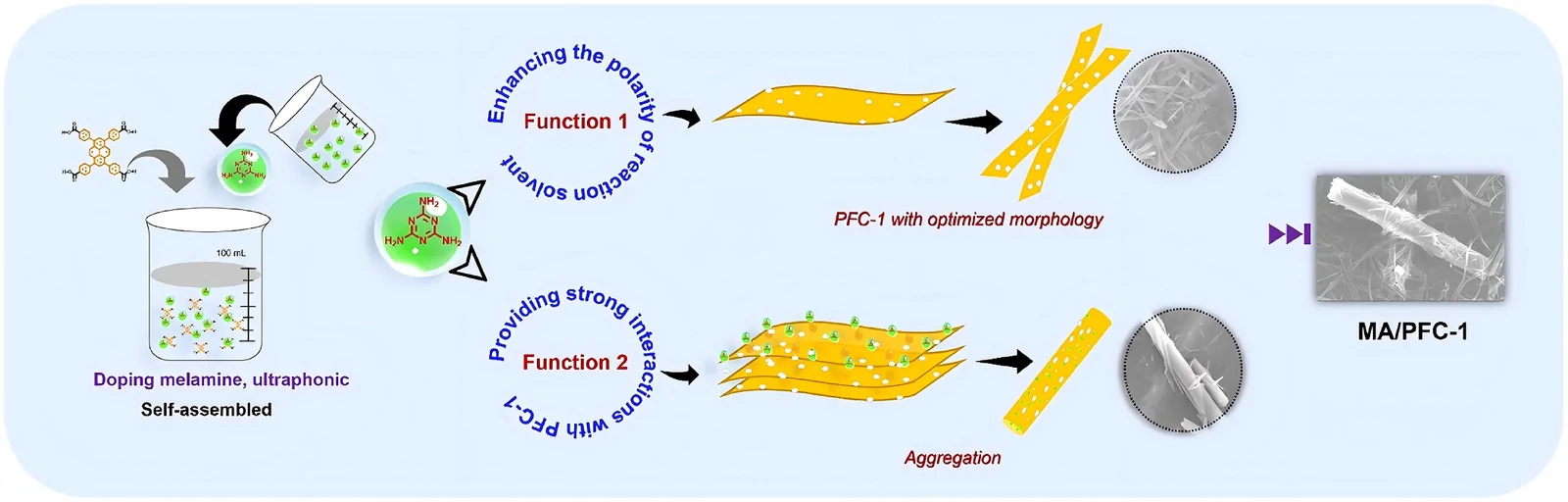
三聚氰胺/1，3，6，8-四（4-羧基苯）芘复合材料的制备示意图^［47］^ MA： melamine.

### 2.4 后合成修饰法

后合成修饰法是先合成具有反应活性基团（如-NH_2_，-COOH）的HOFs母体材料，然后通过后合成修饰反应将功能团（如金属离子、有机分子）引入或锚定到框架上。这种方法可以引入一些无法在直接合成条件下稳定的功能位点。Eu-MOF/HOF复合材料的制备^［46］^实际上包含了一步后合成修饰。首先合成MA-BTC HOF，然后将其与Eu（NO_3_）_3_在*N，N*-二甲基甲酰胺的水溶液中于80 ℃下反应。Eu^3+^离子与HOFs骨架上的羧基等位点配位，在HOFs表面或内部原位形成了Eu-BTC MOFs结构，从而得到了兼具HOFs和MOFs特性的异质结构复合材料，同时保持了原HOFs的主体框架。

HOFs及其复合材料的制备方法丰富多样，从简单温和的溶液法到精确可控的原位生长与化学键合法，不同方法服务于不同的材料设计目标与应用场景。选择合适的方法，关键在于理解并调控分子间弱相互作用与宏观材料性能之间的关系，从而设计出结构稳定、功能优异的HOFs基材料。原位生长法制备的核壳型HOFs复合材料既具有磁分离性能，又具备HOFs高比表面积等特性，为其磁固相萃取应用提供了快速、高效的萃取平台。化学键合法能实现功能基团的稳定固定，可提高材料的机械稳定性，使其能够抵抗色谱分离中流动相的连续冲刷和萃取分离中的多次洗脱。掺杂共组装等功能集成策略，可在分子水平上实现对材料孔径分布和表面化学的精准调控，从而增强HOFs及其复合材料的选择性。

## 3 HOFs材料在分离分析中的应用

HOFs及其复合材料可作为色谱固定相、萃取富集材料和吸附去除材料等应用于色谱分离分析。

### 3.1 HOFs材料作为色谱固定相

氢键的可逆性与多样性使HOFs易于构建具有精密孔道结构和特殊微环境的色谱固定相，从而实现常规材料难以达成的高选择性分离。为进一步阐明HOFs在实际色谱分离中的性能表现，以下将结合GC和CEC中的典型应用案例，详细分析其设计优势与挑战。

#### 3.1.1 气相色谱固定相

Luo课题组^［41］^开创性地利用动态涂覆法将手性材料HOF-2制备成GC固定相。得益于HOF-2固有的、由氢键构筑的手性螺旋孔道（孔径约0.48 nm），该色谱柱不仅成功分离了烷烃、醇类、位置异构体等常规混合物，更在无需衍生的条件下，于2 min内实现了D/L-乳酸等手性分子的基线分离。这充分体现了HOFs结构与功能一体化设计的优势：其温和的溶剂蒸发合成法避免了苛刻条件，且手性微环境直接来自构筑单元的自组装，无需复杂的后修饰。然而，该研究也揭示了HOFs作为固定相稳定性依赖孔径结构的特点，在分析成分复杂的实际血清样品时，由于水、盐分的干扰，其分离效能有所下降，提示HOFs色谱柱在复杂基质中的抗干扰能力仍需提升。

#### 3.1.2 开管毛细管电色谱

CEC要求固定相在液相流动中具备优异的稳定性。早期将纯HOFs直接化学键合用于CEC，虽然实现了对磺胺类药物的良好分离，但多次运行后色谱图出现变化^［45］^。这一局限性正源于纯HOFs仅依靠氢键网络，在连续流动相冲刷下存在结构松动的风险。复合材料的设计为解决上述问题提供了一条有效途径。Ye课题组^［46］^将稀土金属-有机框架与HOFs复合，制备了Eu-MOF/HOF杂化材料，并将其化学键合为固定相。该设计将MOFs的刚性骨架与HOFs的丰富氢键位点巧妙融合，使材料兼具两者优势。MOFs部分主要通过配位键来提升材料的机械稳定性，而HOFs部分不仅提供大量氢键结合位点，还能通过相互作用稳定MOFs结构，拓宽其pH耐受范围。实验结果显示，该复合柱在连续运行100次后，色谱峰依然保持稳定，且柱与柱之间、不同批次间重现性表现优异，保留时间精密度RSD≤0.37%。这一结果表明，通过功能复合策略，HOFs材料成功突破了其在液相色谱应用中长期稳定性的关键瓶颈，为其在复杂分离分析等领域的实际应用铺平了道路。

### 3.2 HOFs作为萃取富集材料

HOFs作为吸附剂的核心优势在于其开放的多孔结构与丰富的弱相互作用位点，可实现快速、高容量的吸附，而其易于功能化的特性使其便于分离操作。

#### 3.2.1 分散固相萃取

分散固相萃取操作简便，通过直接将HOFs吸附剂分散于样品中完成^［40，48］^。Farajzadeh课题组^［40］^利用极其简单的溶剂蒸发法合成了基于1，3，5-苯三甲酸的HOFs，并将其用于分散固相萃取-分散液液微萃取联用技术，成功地从葡萄汁中富集了9种农药。该方法以其制备简便、成本低廉、绿色环保而极具吸引力。HOFs结构中的羧基与农药分子上的特定官能团（如酯、醚、羟基）通过氢键作用，实现了选择性吸附，获得了高达450的富集因子。这展现了HOFs绿色、高效的优势。但此类方法通常依赖离心分离，自动化程度相对较低，在批量样品处理效率上存在局限。

#### 3.2.2 固相微萃取

固相微萃取技术的性能核心在于涂层材料。获得良好的萃取效果需要涂层材料具有高吸附容量、快速传质及优异的稳定性等特性。传统商用涂层（如聚二甲基硅氧烷（PDMS）、聚丙烯酸酯（PA））作用机理单一，在应对理化性质差异显著的多种污染物时存在一定的局限性，比如选择性不足或广谱性差等。为了克服这些问题，Chen课题组^［49］^创新地采用一种高稳定性的氢键有机框架材料PFC-1制备SPME纤维涂层（见图3），并通过一系列实验证明该涂层对于包括多环芳烃、有机氯农药、多氯联苯以及邻苯二甲酸酯在内的多种持久性有机污染物具有良好的富集能力，说明PFC-1作为“通用型”涂层具有突出的优势。其比表面积高达1 235 m^2^/g，紧密的*π-π*堆积和氢键网络支撑使得该材料具有优异的热稳定性，能承受250 ℃高温且可重复利用超过100次。由于孔道结构与多重作用位点（*π-π*作用、氢键、疏水作用）的协同效应，PFC-1展现出广谱吸附能力，它对硝基芳香化合物的富集因子是市场上商用PA纤维的2~11倍，且对于不同种类污染物的检出限低至0.030~0.084 ng/L，在实际样品检测中也表现出良好的重现性与稳定性。这也证明HOFs可用作高性能的SPME涂层，并且可用于复杂基质中广泛存在的污染物的灵敏分析与检测。此外，Chen课题组^［47］^采用掺杂共组装的方法引入三聚氰胺，得到三聚氰胺HOFs（MA/PFC-1）涂层纤维，用于从水样中富集硝基芳香化合物（NACs）。三聚氰胺的存在不仅可以增强氢键，还可以形成分级微孔结构，研究发现，与单纯的PFC-1相比，这种材料对NACs的富集效果更好。这表明可以通过掺杂策略微调HOFs孔道化学，从而获得对特定类别污染物具有更高灵敏度和选择性的材料。

**图3:**
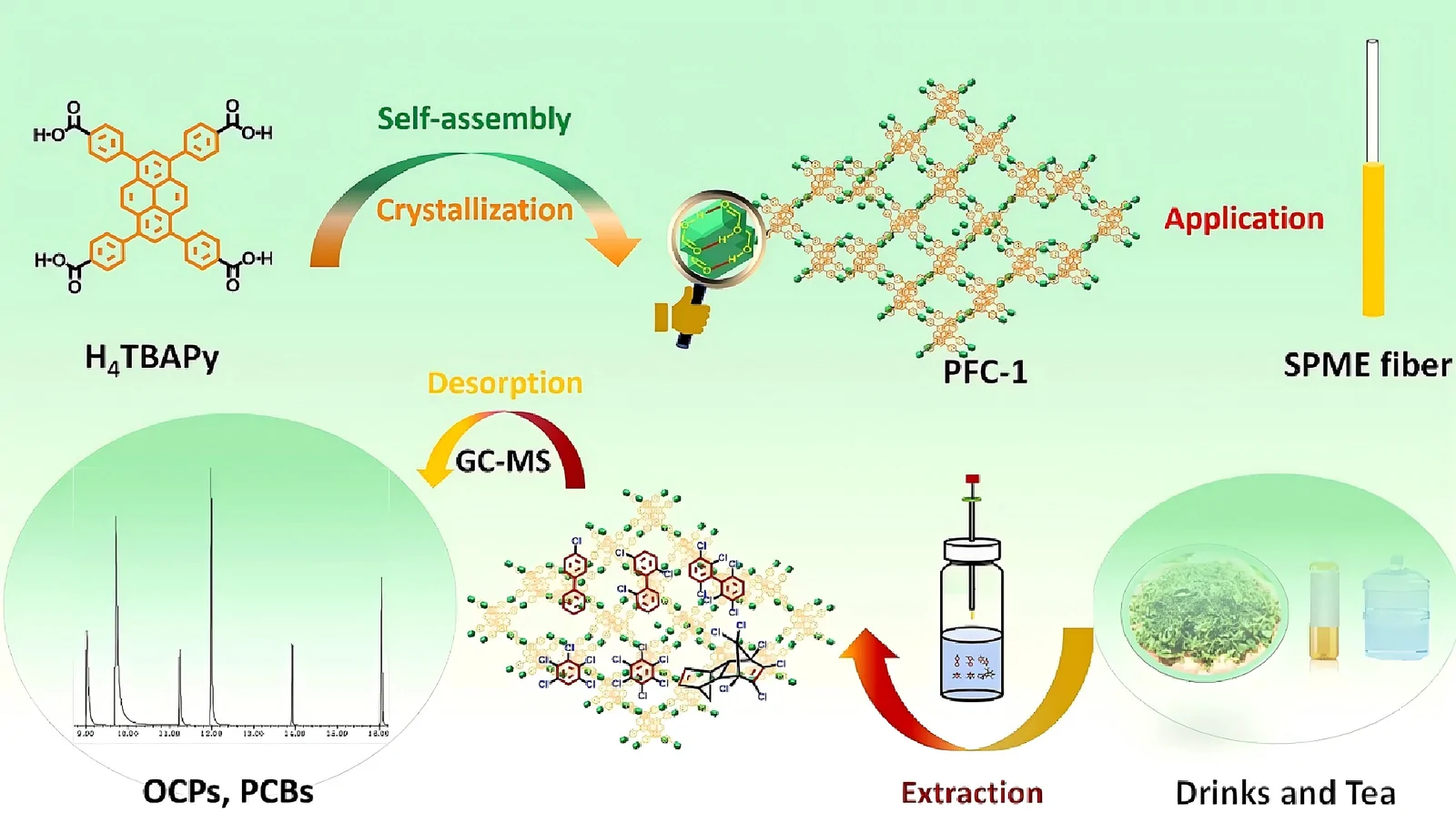
1，3，6，8-四（4-羧基苯）芘纤维的制备及其在GC-MS联用分析有机氯农药和多氯联苯中的应用示意图^［49］^ H_4_TBAPy： 1，3，6，8-tetra（4-carboxylphenyl）pyrene； OCPs： organochlorine pesticides； PCBs： polychlorinated biphenyls； GC-MS： gas chromatography-mass spectrometry； SPME： solid phase microextraction.

#### 3.2.3 磁固相萃取

核壳型磁性HOFs复合材料既具有HOFs的高比表面积、选择性吸附位点，又具有Fe_3_O_4_的磁性，在外加磁场下可实现磁性HOFs复合材料吸附剂的快速、无损分离和回收利用，有效简化操作步骤，提高样品前处理效率。Lu课题组^［50］^采用液相共混法合成了具有介孔结构的Fe_3_O_4_@PFC-1复合材料，其表面富含苯环和羧基等活性基团，能提供氢键、*π-π*相互作用等多种相互作用力，可在10 s内实现多种双酚类化合物的吸附。在真实环境水样、土壤以及生物样本分析中，该吸附剂展现出高富集因子（234~296），且可以稳定循环使用13次以上。我们课题组^［44］^采用原位自组装方法制备了具有均匀核壳结构的Fe_3_O_4_@HOFs材料，用于食品样品中四环素的萃取和检测分析。该吸附剂用量仅为2 mg，5 min即可达到吸附平衡，结合HPLC-DAD检测，检出限低至0.3 ng/mL。分子模拟等实验结果证明其特异性识别四环素的原因是氢键、*π-π*堆积及疏水作用的协同作用，吸附剂经过8次重复使用后性能依旧稳定。由此可见，磁性HOFs复合材料具备超快分离、优异吸附动力学、高选择性和良好的可循环性优势，是磁固相萃取领域极具前景的新型吸附材料。但是这类材料的性能很大程度上受到HOFs壳层的均匀性和稳定性影响，在不同批次间保证材料性能的高度重现性是其走向商业化过程中面临的一大难题。

### 3.3 HOFs作为吸附去除材料

HOFs因其高比表面积和丰富的表面官能团，可直接用作吸附剂去除水体中的染料、重金属离子和放射性核素等污染物。

#### 3.3.1 重金属吸附

Wang课题组^［51］^对多功能氢键有机框架平台PFC-1进行了研究和报道。该平台能同时选择性地实现对重金属离子Pb^2+^和Cu^2+^的荧光传感检测与高效吸附去除。在传感器领域，PFC-1利用荧光猝灭效应对Pb^2+^和Cu^2+^进行检测，展现出优异的检测性能，检出限分别为2.6 µmol/L和4.5 µmol/L。作为吸附剂，PFC-1对Pb^2+^的最大吸附容量高达384.6 mg/g，对Cu^2+^的最大吸附容量为214.1 mg/g，且在约30 min内达到吸附平衡，材料性能可与多种MOFs/COFs相媲美。研究揭示了传感与吸附的内在机制：静电相互作用、离子交换以及重金属离子与PFC-1羧基位点的配位作用（螯合）是吸附的主要驱动力。导致荧光猝灭的关键在于配位引发的主客体电荷转移。材料经多次吸附-脱附循环后仍保留较好的性能，证明了其应用的稳定性和可行性。该研究实现了检测与去除一体化，为污染物的原位监测与同步治理提供了创新思路。

#### 3.3.2 放射性核素吸附

目前放射性核素吸附很好地体现了HOFs的易功能化特性，可根据放射性核素性质定向设计HOFs结构，实现放射性核素的高效、选择性吸附。在铀酰离子吸附方面，研究者们设计和制备了具有高选择性的HOFs材料，实现了铀酰离子的高效分离与富集。Liu课题组^［42］^合成了八重互锁结构的HOF-BTB（见图4）。这种致密、互锁的坚固骨架在水相介质，甚至酸性或碱性溶液中均显示出很好的结构稳定性，循环使用3次后HOF-BTB材料对铀酰离子的吸附效率仍能达99.7%，而且可实现溶液中99.8%的铀去除（铀酰离子初始质量浓度为5 µg/mL）。因HOF-BTB材料的互锁结构和丰富的羧基位点，HOF-BTB吸附容量高达451 mg/g，结合荧光检测方法，可实现铀酰离子的高灵敏检测，检出限低至2.35 µg/L。Li课题组^［52］^设计了一种磷酸功能化的HOFs材料（P-HOF），在宽pH范围（1~9）和真实海水中均表现出优异的稳定性。通过P=O和P-O基团对铀酰离子UO_2_^2+^的配位作用，P-HOF的吸附容量高达1 066 mg/g。HOF-BTB利用优化的互锁框架拓扑结构提升稳定性，并提供丰富的羧基吸附位点；而P-HOF则在分子层面上通过后修饰高选择性磷酸功能基团实现对UO_2_^2+^的配位吸附。虽然在设计策略上各有侧重，但它们在极具挑战性的海水提铀方面效果优异，共同体现了HOFs在放射性核素分离领域的巨大潜力。

**图4:**
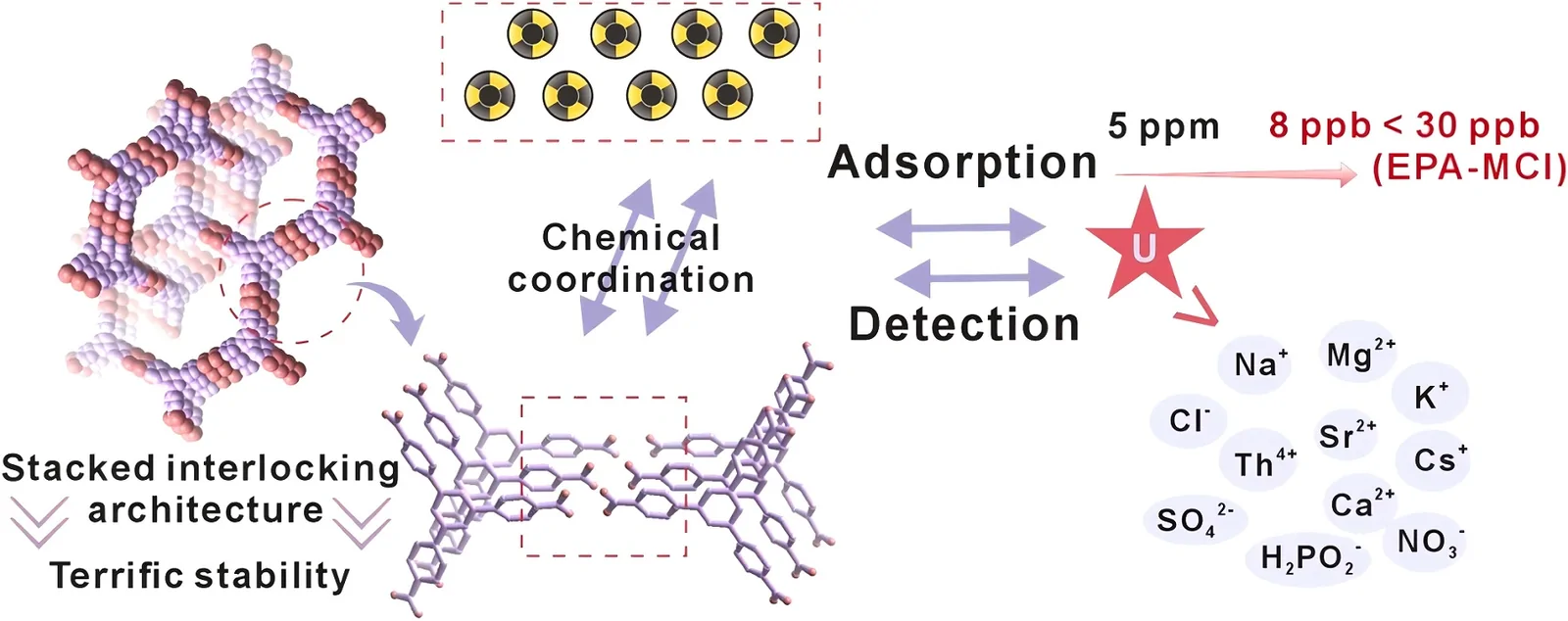
八重互锁结构的HOF-BTB高效去除和灵敏检测铀酰离子示意图^［42］^ EPA-MCI： the United States Environmental Protection Agency-the maximum contaminant level for uranium.

Wang课题组^［43］^报道了一种能够在常温常压下实现克级规模合成的单晶HOFs材料H1。该材料展现了顶尖的碘吸附性能：在75 ℃常压蒸气吸附中，其平衡吸附量达6.02 g/g，动力学符合准二级模型，300 min内即可完成吸附。在水相中，H1对1.0 mmol/L碘溶液的去除率高达97.2%，5 min内即可吸附约65%的碘；在饱和KI/I_2_溶液中，其吸附容量高达4.44 g/g，为目前报道的HOFs材料中水相碘吸附的最高纪录。更重要的是，研究开创性地实现了吸附后材料的资源化应用：将碘负载后的材料I_2_@H1直接用作碘源用于合成*β*-碘代醚，产率与使用商业单质碘原料相当。机理研究表明，碘吸附过程包含初始的物理捕获与后续的化学吸附，碘分子进入框架后发生电子转移形成I_3_⁻等多碘离子。吸附的碘可在甲醇中高效释放，8 h内释放率>94%。此外，材料展现出优异的稳定性，其在多种有机溶剂、碱液甚至冰醋酸中均保持稳定。该研究突破了高质量单晶HOFs难以规模化合成的瓶颈，并开创了从“吸附去除”到“资源化利用”的应用新范式，气相和水相碘吸附性能均处于世界领先水平，为HOFs在放射性碘捕获及资源循环领域展示了独特价值。

#### 3.3.3 有机染料吸附

Hao课题组^［53］^系统探究了氢键有机框架材料 PFC-1对水中有机染料的吸附性能。研究表明，PFC-1对阳离子染料罗丹明B（RhB）和阴离子染料甲基橙（MO）均展现出优异的吸附能力，在298.15 K时的最大吸附容量分别高达317 mg/g和252 mg/g。通过热力学研究得到RhB的吸附过程吸热，而MO的吸附过程放热。结合各项机理表征分析，PFC-1材料的羧基和苯环主要通过氢键、*π-π*作用、范德华力和静电吸引等作用有效捕获染料分子。其中，对RhB的吸附主要依赖于静电作用、氢键和*π-π*相互作用，对MO的吸附主要依赖于范德华力。PFC-1对RhB的吸附去除率在5次重复使用后仍能达到99%，证明该材料具有优异的重复利用性和稳定性。理论研究往往是在简单体系中进行，而实际的工业废水中往往非常复杂，常有多种染料、无机盐离子和其他污染物共存，在这种复杂体系中会有不可避免的非特异性吸附和竞争吸附，因此在今后的研究中应提高HOFs材料的选择性吸附和共吸附能力。对于阳离子染料，可功能化磺酸基团，提高HOFs的选择性吸附能力；对于阴离子染料，则可功能化季铵盐基团；同时可结合分子尺寸效应、静电作用、pH响应或光响应等综合开发多种协同机制共吸附HOFs材料。

## 4 结论

作为一种新兴多孔晶体材料，HOFs因制备条件温和、结构可控、易功能化等特点，在分离分析领域展现出一定的应用潜力。目前可通过溶剂挥发、溶剂热及自组装等方法制备HOFs，并通过原位生长、化学键合、掺杂共组装或后修饰获得HOFs功能复合材料。这些材料已成功用作色谱固定相、固相萃取吸附剂以及重金属、放射性核素、有机染料的高效吸附去除材料。然而，HOFs的孔径、比表面积等结构性质与其吸附选择性、吸附容量和吸附动力学等吸附性能的构效关系还需要进一步探究。未来需要依据分离分析需求，理性设计和优化制备具有高比表面积、可调孔径和丰富官能团的高效、高选择性HOFs材料，并将其与微流控芯片等微型化设备融合，提高自动化分离分析水平。
